# Veterinary World reviewer acknowledgment 2024

**DOI:** 10.14202/vetworld.2025.249-251

**Published:** 2025-01-30

**Authors:** A.V. Sherasiya, Riyaz

**Affiliations:** Veterinary World, Star, Gulshan Park, NH-8A, Chandrapur Road, Wankaner - 363621, Dist. Morbi, Gujarat, India

## Contributing reviewers

Veterinary World editorial team sincerely like to thank all of our reviewers who contributed to peer review for the journal in 2024.

**Table:** 

No.	Reviewer	Country	Reviewed articles
1	A. Aygun	Turkey	4
2	A. K. Bulashev	Kazakhstan	1
3	Aamir Ali	Pakistan	1
4	Abhijit S. Deshmukh	India	1
5	Abolfazl Ghaniei	Iran	1
6	Agung Irawan	USA	1
7	Ahmed S Abdoon	Egypt	1
8	Ahmed Saad Ahmed Hassaneen	Egypt	1
9	Alberto Elmi	Italy	1
10	Alejandro Hidalgo	Chile	2
11	Alfredo Medrano	Mexico	2
12	Ali Anil Suleymanoglu	Turkey	2
13	Alireza Seidavi	Iran	1
14	Alvaro DiazBadillo	USA	1
15	Amira Taman	Egypt	1
16	Amitava Dey	India	1
17	Amjad Islam Aqib	Pakistan	1
18	Anas Abdullah Hamad	Iraq	5
19	Andreas Mershin	USA	1
20	Andreia Coutinho Facin	Brazil	1
21	Antonio J. Vallecillo	Ecuador	1
22	Anut Chantiratikul	Thailand	2
23	Arda Sozcu	Turkey	3
24	Artem Blagodatski	Russia	1
25	Ashok kumar	India	1
26	Ashwani Kumar	India	2
27	Asim Faraz	Pakistan	1
28	Ayman Abdel-Aziz Swelum	Egypt	1
29	Ayrton Breno Pimenta	Brazil	1
30	Aysu Deirmenci Dkaya	Turkey	1
31	Banan Khalid Al-Baggou	Iraq	1
32	Baratang Alison Lubisi	South Africa	2
33	Batoul Mohamed Izzularab	Egypt	1
34	Belal S Obeidat	Jordan	1
35	Belgin Siriken	Turkey	1
36	Bernad, Elena	Romania	1
37	Blazej Nowak	Poland	2
38	Brandon Flatgard	USA	1
39	Breno Fernando Martins de Almeida	Brazil	1
40	Buket Er Demirhan	Turkey	2
41	Bulent Ekiz	Turkey	1
42	C. Usugamonroy	Colombia	2
43	Carmen Solcan	Romania	2
44	Cassia Regina Oliveira Santos	Brazil	1
45	Cenk Er	Turkey	1
46	Chao-Nan Lin	Taiwan	1
47	Charleata A Carter	USA	3
48	Cher Chien Lau	Malaysia	1
49	Christin Kleinsorgen	Germany	1
50	Clarissa Silveira Luiz Vaz	Brazil	1
51	Claudia Giannetto	Italy	1
52	Csaba Hancz	Hungary	1
53	Dariusz Kucharczyk	Poland	1
54	Debasis Nayak	India	1
55	Deepak Gola	India	1
56	Deepan Kishore	USA	1
57	Dhruv Nitinkumar Desai	USA	1
58	Di Cataldo Sophia	Argentina	2
59	dilip kumar swain	India	1
60	Dimar Sari Wahyuni	Indonesia	1
61	Dmitrii E. Aleshin	Russia	1
62	Nadine Ndilimabaka	Gabon	1
63	Anupama Dr. Mukherjee	India	1
64	Archana Jain	India	1
65	Bhanusupriya	USA	1
66	Pourouchottamane R	India	2
67	V.V.S.Suryanarayana	India	1
68	E Uzun Yaylac	Turkey	1
69	E.E. Onbaşilar	Turkey	1
70	Edson Ireeta Munanura	Uganda	1
71	Eduardo Ghiggi	Brazil	1
72	Eduardo Jorge Boeri	Argentina	4
73	Elena Garde	Chile	1
74	Elif Çelik	Turkey	2
75	Elise L. McKean	USA	1
76	Elizabeth SalinasEstrella	Mexico	1
77	Eloiza May Galon	Philippines	1
78	Eman Dhahir Arif	Iraq	1
79	Eman S. ElWakil	Egypt	2
80	Emily Haddy	United Kingdom	1
81	Essam Almadaly	Egypt	2
82	Ethayathulla Abdul Samath	India	1
83	Ewa SellKubiak	Poland	1
84	Fabrizio Bertelloni	Italy	2
85	Fatma M.A. Yousseff	Egypt	1
86	Fei Zhao	China	3
87	Fernando de Freitas Fernandes	Brazil	1
88	Firmain N. Yokoly	Cote D’Ivoire	1
89	Florence D Tarfa, David Tarfa	Nigeria	1
90	Fouad Kasim Mohammad	Iraq	6
91	Francesco Serrapica	Italy	1
92	Gabriel Vieira Ramos	Brazil	1
93	Gautham Kolluri	India	1
94	Genevieve Andre-Fontaine	France	2
95	Ghadeer Sabah Bustani	Iraq	3
96	Gianmarco Ferrara	Italy	3
97	Hani H. AlBaadani	Saudi Arabia	1
98	Hany Elsheikha	UK	1
99	Harvie P. Portugaliza	Philippines	2
100	Hasan Aydin	Turkey	1
101	Hasan Meydan	Turkey	4
102	Hasria Alang	Indonesia	2
103	Haval Mohammed Khalid	Iraq	1
104	Haytham Senbill	Egypt	1
105	Hazem Shaheen	Egypt	3
106	Hector Gabriel Varela	Argentina	1
107	Heliot Zarza	Mexico	1
108	HuaJi Qiu	China	3
109	Hui-Chen Guo	China	1
110	Hung Luong	USA	4
111	Iang Schroniltgen Rondon Barragan	Colombia	1
112	Ilona Kaszak	Poland	1
113	Indrasen Chauhan	India	3
114	Ionel Alexandru Checherita	Romania	1
115	Ionel D. Bondoc	Romania	1
116	Ismael Hernandez Avalos	Mexico	2
117	İsmail Hakkı Tekiner	Turkey	1
118	Ivo Sirakov	Bulgaria	1
119	Jabulani Nkululeko Ngcobo	South Africa	1
120	Jamal Gharekhani	Iran	1
121	Jareerat Aiemsaard	Thailand	4
122	Jianye Wang	China	1
123	JiunLing Wang	Taiwan	1
124	Jnos Dgi	Romania	3
125	João Simões	Portugal	4
126	Joaquin Gadea	Spain	1
127	John F. Mee	Ireland	1
128	Joice San Andres	Philippines	1
129	Jonathan T Bvunzawabaya	USA	1
130	Jose Francisco Rivera Benitez	Mexico	1
131	Jose Luis Campos	Spain	1
132	Joshua Mbanga	Zimbabwe	1
133	Julian Ruiz Saenz	Colombia	1
134	Kannan Thandavan Arthanari	India	2
135	Karim El Sabrout	Egypt	5
136	Kavous Solhjoo	Iran	1
137	kekungu puro	India	1
138	Kezhou Cai	China	1
139	KimDiep Tran	Vietnam	2
140	Krpasha Dixon	United Kingdom	2
141	Laurent F. Bonnac	USA	1
142	Lawrence Mugisha	Uganda	1
143	Lenin Lenin Aguirre Riofrio	Ecuador	2
144	Letizia Sinagra	Italy	1
145	Liga Kovalcuka	Latvia	2
146	Ligi Milesh	USA	4
147	Liliana Aguilar-Marcelino	Mexico	1
148	Linous Munsimbwe	Zambia	2
149	Lonneke L. IJsseldijk	Netherlands	1
150	Luca Todini	Italy	1
151	M Saminathan	India	1
152	M Zamri Saad	Malaysia	1
153	M. Teresa Teresa Tejedor	Spain	1
154	Mahipal Singh Sankhla	India	1
155	Mahmoud Mohey Elhaig	Egypt	1
156	Majid Fasihi Harandi	Iran	1
157	Malgorzata Gajewska	Poland	1
158	María Denisse Montoya-Flores	Mexico	1
159	Maria Florencia Gallelli	Argentina	1
160	Maria Paola Cassarani	Italy	1
161	Mariola Bochniarz	Poland	1
162	Martina Gerken	Germany	1
163	Maryam Dadar	Iran	2
164	Md. Zakir Hassan	Bangladesh	1
165	Md. Ahaduzzaman	Bangladesh	4
166	Md. Tanvir Rahman	Bangladesh	1
167	Megha Agarwal	USA	2
168	Miguel Tavares Tavares Pereira	Switzerland	1
169	Mihaela Niculae	Romania	4
170	Mirosław Mariusz Michalski	Poland	3
171	Mohamed Abomosallam	Egypt	1
172	Mohammed Baqur S AlShuhaib	Iraq	1
173	Mohammed El-Houadfi	Morocco	1
174	Mohammed S. ALhajj	Saudi Arabia	2
175	Mohan Singh Thakur	India	1
176	Mona Ahmed ElZamkan	Egypt	1
177	Mona Said Mahmoud	Egypt	1
178	Morakot Kaewthamasorn	Thailand	1
179	mosua tavassoli	Iran	1
180	MR Marques	Portugal	1
181	Muhammad Abdul Basit	Pakistan	2
182	N. W. Khalil	Egypt	1
183	Nalinee Tuntivanich	Thailand	2
184	Nantawan Therdthai	Thailand	1
185	Nataliia Mokhnachova	Ukraine	1
186	Natapol Pumipuntu	Thailand	1
187	Nesreen Allam Tantawy Allam	Egypt	1
188	NiLuh Putu Dharmayanti	Indonesia	1
189	Noha Mahmoud Fahmy Hassan	Egypt	1
190	Noppason Pangprasit	Thailand	1
191	Nur Azira Tukiran	Malaysia	2
192	Nurliyana Mohamad	Malaysia	1
193	Om Prakash Choudhary	India	2
194	Panagiotis E Simitzis	Greece	7
195	Paulraj Philip Samuel	India	1
196	Peerapol Sukon	Thailand	2
197	Piya Temviriyanukul	Thailand	1
198	Pınar Peker Akalın	Turkey	3
199	Pınar Şanlıbaba;	Turkey	2
200	Prof ElSayed Eldeeb	Egypt	10
201	QiZhong Zhang	China	2
202	R. Rajasekhar	India	1
203	Rafael Cardoso Carvalho	Brazil	1
204	Rajeev Ranjan	India	1
205	Rajendran K. V.	India	1
206	Rao Zahid Abbas	Pakistan	1
207	Rasa Binkien	Lithuania	1
208	Reenu Singh Tanwar	USA	1
209	Ricardo Tafuri Brando	Brazil	1
210	Riyaz Sherasiya	India	4
211	Robert J. Geraghty	USA	1
212	roberta perego	Italy	1
213	Rodrigo Alberto Jerez Ebensperger	Spain	4
214	Rukhsana Amin Runa	Bangladesh	1
215	S.K. Mondal	India	2
216	Saber Raeghi	Iran	1
217	Sachin Kumar	India	1
218	Saganuwan Alhaji Saganuwan	Nigeria	1
219	Sajid Ali	Pakistan	1
220	Sakshaleni Rajendiran	Malaysia	1
221	Salvador Eduardo AcevedoMonroy	Mexico	1
222	Sana A M Elgayar	Egypt	1
223	SanYuan Huang	Taiwan	1
224	Saran Promsai	Thailand	3
225	Saravanan Subramaniam	India	2
226	Saw Bawm	Myanmar	1
227	Seyedeh Mahdieh khoshnazar	Iran	1
228	Shahid Nazir	Australia	1
229	Shaimaa H. Negm	Egypt	2
230	Shameeran Salman Ismael	Iraq	2
231	Shekhar Ramchandra Bhagwat	India	4
232	ShengFan Wang	Taiwan	1
233	Siju Susan Jacob	India	1
234	Simon Peter Musinguzi	Uganda	1
235	Sintija Jonova	Latvia	4
236	sleyman ilek	Turkey	7
237	Socheat Sieng	Australia	1
238	Sorawat Thongsahuan	Thailand	1
239	Suchawan Pornsukarom	Thailand	1
240	Sucheeva Junnu	Thailand	1
241	Sudhakar Pralhad Awandkar	India	1
242	Sumera Sajjad	Pakistan	1
243	Suresh H Basagoudanavar	India	1
244	Takalani Judas Mpofu	South Africa	2
245	Tanko Polycarp Nwunuji	Nigeria	2
246	ThankGod E. Onyiche	Nigeria	1
247	Thotsapol Thomrongsuwannakij	Thailand	2
248	Todd Hsu	Taiwan	1
249	Tong Bu	China	1
250	Toshiro Arai	Japan	1
251	Trker Sava	Turkey	1
252	Usama T. Mahmoud	Egypt	1
253	Usman Ibe Michael	Uganda	1
254	Valeria Silva Alvarez	Uruguay	1
255	Vanessa S. Cruz	Brazil	2
256	Vasfiye Kader Esen	Turkey	1
257	Vicente RodrguezEstvez	Spain	1
258	Victor Alves Carneiro	Brazil	1
259	Vijaya Kumar Anumolu	India	1
260	Vimbai Gobvu	Zimbabwe	1
261	Vincenzo Tufarrelli	Italy	1
262	Vishal S Suthar	India	3
263	Vukka Chengalva Rayulu	India	1
264	Wael A. Khalil	Egypt	1
265	Weerapol Taweenan	Thailand	1
266	Widya Paramita Lokapirnasari	Indonesia	4
267	Wisnu Nurcahyo	Indonesia	1
268	Xiaole Qi	China	1
269	Yetti Marlida	Indonesia	1
270	Yun Ma	China	1
271	Zahid Naseer	Pakistan	1
272	Zaven Karalyan	Armenia	1
273	Zeki Erol	Turkey	1
274	Zhiyong Fan	China	1

